# Elucidating the cellular dynamics of the brain with single-cell RNA sequencing

**DOI:** 10.1080/15476286.2020.1870362

**Published:** 2021-01-27

**Authors:** Aida Cardona-Alberich, Manon Tourbez, Sarah F. Pearce, Christopher R. Sibley

**Affiliations:** a Institute of Quantitative Biology, Biochemistry and Biotechnology, School of Biological Sciences, Edinburgh University, Edinburgh, UK; b Simons Initiative for the Developing Brain, University of Edinburgh, Edinburgh, UK; c Centre for Discovery Brain Sciences, University of Edinburgh, Edinburgh, UK; d Euan MacDonald Centre for MND Research, University of Edinburgh, Edinburgh, UK

**Keywords:** Single-cell, single-nuclei, scRNA-seq, snRNA-seq, neurodegeneration, neurodevelopmental disorder

## Abstract

Single-cell RNA-sequencing (scRNA-seq) has emerged in recent years as a breakthrough technology to understand RNA metabolism at cellular resolution. In addition to allowing new cell types and states to be identified, scRNA-seq can permit cell-type specific differential gene expression changes, pre-mRNA processing events, gene regulatory networks and single-cell developmental trajectories to be uncovered. More recently, a new wave of multi-omic adaptations and complementary spatial transcriptomics workflows have been developed that facilitate the collection of even more holistic information from individual cells. These developments have unprecedented potential to provide penetrating new insights into the basic neural cell dynamics and molecular mechanisms relevant to the nervous system in both health and disease. In this review we discuss this maturation of single-cell RNA-sequencing over the past decade, and review the different adaptations of the technology that can now be applied both at different scales and for different purposes. We conclude by highlighting how these methods have already led to many exciting discoveries across neuroscience that have furthered our cellular understanding of the neurological disease.

## 1. Introduction

The central nervous system (CNS) is a remarkably complex tissue composed of billions of highly differentiated and interconnected cells of various lineages. This includes a plethora of neuronal cells that form intricate neural networks directing physical activities and high-level cognitive functions (e.g. decision-making, memory, social behaviour). The numerous neuroglia then perform a wide array of trophic functions to support this complex environment. Among others, microglia are the resident macrophages of the CNS that provide it with a form of active immune defence, oligodendrocytes provide supporting myelin sheaths around neuronal axons that facilitate rapid propagation of electrical signals, and astrocytes provide biochemical support to surrounding cells and control the extracellular environment at synapses to help regulate the transmission of electrical signals between neurons.

This cellular diversity and intricate connectivity mean that a complex interplay occurs between many cell types during neurodevelopment, normal CNS function and the manifestation of the various diseases of the CNS. Accordingly, in order to further our understanding of the CNS in health and disease the field requires enhanced knowledge of this complex system at the cellular level. However, this interplay has been challenging to address using our long-standing technical abilities to monitor cell morphology, neuroanatomy, electrophysiology, synapse biology and neural connectivity that have provided so much of our current knowledge of the CNS.

Excitingly then, the development of single-cell RNA-sequencing (scRNA-seq) and several closely related technologies now offer an unprecedented opportunity to interrogate the intricate cellular dynamics of the CNS at cell-type-specific resolution through the study of RNA metabolism. Indeed, the RNA transcriptome of an individual cell can readily identify a cell’s background whilst concomitantly providing a remarkably accurate snapshot of how the cell is both functioning and responding to external cues at the time of profiling. Moreover, it has the ability to tease apart the cellular heterogeneity in seemingly homogenous cell populations that may offer explanations to the emergence of certain complex phenotypes. With this in mind, in this review, we discuss the technological principles underlying this emerging field, then present the early and exciting applications of scRNA-seq that have emerged across the neurosciences.

## 2. From single cells to scRNA-seq datasets

ScRNA-seq remains a relatively new technology to many, yet it has its origins in the pioneering work of both Norman Iscove and James Eberwine in the early 1990s. In their seminal experiments, the RNA from single cells was reverse transcribed and exponentially amplified with PCR [1,2], or linearly amplified with *in vitro* transcription (IVT) [3]. The resulting amplification of the individual cellular transcriptomes could subsequently be used as a template for PCR or southern blots against genes-of-interest. The advent of microarray technology and next-generation sequencing (NGS) subsequently catapulted the field to the transcriptome-wide analyses that are becoming increasingly frequent today, with NGS now the preferred of these options. Excitingly for the field, scRNA-seq has experienced a revolution in recent years following the introduction of numerous technical modifications which have made the protocol easier, faster, more reliable, and capable of profiling ever-increasing numbers of cells (Table 1). Indeed, the limiting factor for single-cell RNA-seq studies typically remains the cost of the sequencing itself.

**Table 1.** Key scRNA-seq and snRNA-seq method adaptations

Method	Cells	Concept	Advantages	Disadvantages	Reference
**STRT-seq** (single-cell tagged reverse-transcription sequencing)	1 × 10^2^	Indexed template-switching oligos used for RT-based barcoding of cells in individual wells	First barcoding strategy allowing for multiplexing of multiple cells Multiplexing minimizes well-to-well technical biases	5ʹ bias Manual single cell isolation	^[4]^
**CEL-seq** (Cell Expression by Linear amplification and sequencing)	5 × 10^2^	Barcoded RT primers allows early pooling before IVT amplification	Linear amplification preserves relative abundances of mRNA transcripts Detects more genes than STRT-seq Higher sensitivity than STRT-seq	3ʹ bias High-abundance transcripts bias Manual single cell isolation	^[7]^
**SMART-seq** (Switching Mechanism at 5ʹ End of RNA Template)	1 × 10^2^	Full-length cDNA amplified following a template-switching reverse transcription reaction	Full-length cDNA Allows splice isoforms to be discerned	Lack of strand specificity Selective for polyadenylated RNA Increased labour compared to methods with early multiplexing	^[13]^
**SMART-seq2** (Switching Mechanism at 5ʹ End of RNA Template)	10^2^–10^3^	As above	As above Increased cDNA yield from single cells, higher sensitivity, fewer technical biases, less variability, and less expensive compared to SMART-seq Initial steps are used by many scRNA-seq workflows	As above Transcript length bias (inefficient with mRNAs > 4 Kb) High-abundance transcript bias	^[14]^
**SMART-seq3**	10^2^–10^3^	As above but with UMI incorporated into template-switching oligo for *in silico* isoform analysis.	Optimized steps leads to improved cDNA yields and library complexity Strand specific Improved ability to discern isoforms and allelic expression	As above Transcript length limitation to *in silico* isoform analysis	^[15]^
**Quartz-seq/Quartz-seq2**	10^2^–10^4^	Poly-A tailing and 2^nd^ strand synthesis instead of template-switching oligo for full-length transcript amplification	Highly reproducible cell transcriptomes Increased library complexity leads to reduced sequencing requirements High UMI conversion rate for quantitative studies.	High mRNA GC content reduces detection 3ʹ bias Bias towards shorter transcripts	^[18,132]^
**Fluidigm C1**	10^2^–10^3^	Individual cell capture and library processing in commercially available integrated microfluidics circuits	Automated processing minimizes technical bias Full length sequencing Circuits optimized for different cell sizes	Specialist equipment required	^[6]^
**MARS-Seq** (massively parallel single-cell RNA-sequencing)	> 10^3^ cells	Automated, multi-step barcoding of cells FACS sorting of cells into well, three levels of barcoding	Automatization of single cell isolation with FACS Increased throughput possible High degree of multiplexing	Requires specialist equipment (FACS, liquid handler) Lack of strand specificity 3ʹ bias	^[12]^
**Droplet based scRNA-seq** (i.e. Drop-seq/inDrops)	10^3^–10^6^	Microfluidics capture of cells in oil droplets, RT based barcoding i) on mRNA capture beads (Drop-seq) or ii) in droplets (inDrops)	Parallel processing of large number of cells for increased scalability at reduced cost	3ʹ bias Requires custom microfluidics system Low mRNA capture efficiency with inDrops	^[22,43]^
**SPLiT-seq** (Split-pool ligation-based transcriptome sequencing)	> 10^5^	*In situ* barcoding of RNA via multiple sequential split-pool reactions	Compatible with fixed cells or nuclei Efficient sample multiplexing No customized equipment required Avoids non-trivial single cell isolation/sorting	Labour intensive Low complexity per cell in current studies 3ʹ bias	^[24]^
**sci-RNA-seq** (single-cell combinatorial indexing RNA sequencing)	5 × 10^4^	*In situ* mRNA barcoding followed by second barcoding step during PCR amplification	Compatible with fixed cells or nuclei Efficient sample multiplexing Scalable via extra tagmentation barcoding, or use of 384-well plates FACS step helps eliminate doublets	Labour intensive 3ʹ bias	^[46]^
**Scifi-seq** (single cell combinatorial fluidic indexing)	10^3^–10^6^	*In situ* pre-indexing of cell transcriptomes followed by scRNA-seq with droplet based sequencing	Multiple cells per droplet increases cell/nuclei throughput ~15-fold. Easy multiplexing Faster than multi-round combinatorial indexing Efficient reagent use and sequencing costs High complexity per cell	Non-trivial optimization 3ʹ bias Requires custom microfluidics system	^[47]^
**Pico-well based sequencing**	>10^4^	PDMS-based printing of >10,000 pico-wells to facilitate the gravitational capture of individual cells and mRNA capture beads in high throughput	Cost-effective Readily scalable Recent versions improve cell separation and reduce cross-contamination	Requires custom fabricated chips 3ʹ bias	^[40]^
**snRNA-seq** (i.e. SNS, sNuc-seq)		Single nuclei used instead of whole cell SNS uses fluidigm C1 system sNuc-seq uses Smart-seq2	Study of difficult to dissociate cell types (e.g. neural tissue, archived tissue) Full length sequencing	Low mRNA capture due to rapid nuclear export following poly-adenylation Missing information from cytoplasmic transcriptome FACS dependent	^[58,133]^
**Droplet based snRNA-seq** (i.e. DroNc-seq, snDrop-seq)	5 × 10^4^	Massively parallel single nuclei profiling with droplet technology	Study of difficult to dissociate cell types (e.g. neural tissue, archived tissue) Parallel processing of large number of nuclei for increased scalability at reduced cost	Low mRNA capture due to rapid nuclear export following poly-adenylation Missing information from cytoplasmatic transcriptome 3ʹ bias	^[23,57]^

**Figure 1.**
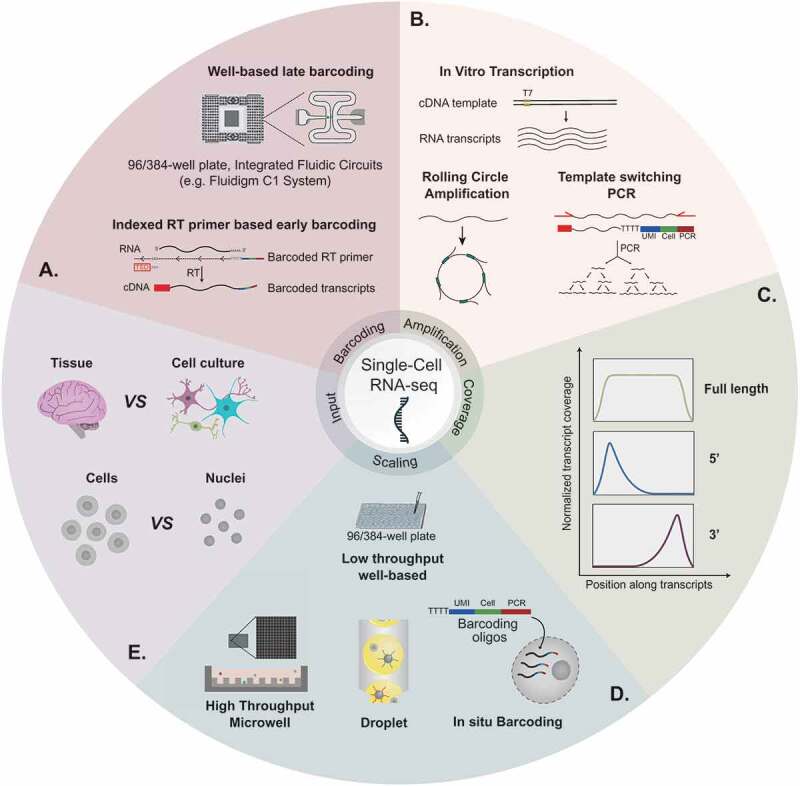
**A recipe book for scRNA-seq: A)** Cellular barcoding can be achieved by PCR or tagmentation-based late indexing of independent cDNA libraries prepared in separated chambers, or via early introduction of cell barcodes during the reverse transcription reaction. **B)** Amplification of low abundance cellular material can be achieved by in vitro transcription, PCR following a template-switching reverse transcription reaction, or rolling circle amplification. **C)** The choice of scRNA-seq workflow will determine the distinct coverage enrichment profiles observed along captured RNA transcripts. **D)** Independent cDNA libraries prepared in separated chambers can lead to full transcript coverage at reduced scale. Increased scaling can be achieved by increasing the number of ‘chambers’ from which libraries are prepared, and providing cellular indexes during reverse transcription such that early pooling of cellular transcriptomes can be achieved. The former can be achieved using massively parallel microwells, using microfluidic systems to rapidly generate thousands of oil droplet-based reaction chambers that encapsulate single cells, or employing the cells themselves as the reaction chambers for *in* *situ* library preparations. **E)** Starting material can be heterogeneous tissue or cell culture preparations. The characteristics of the input will determine whether whole cells or purified nuclei are to be profiled

**Figure 2.**
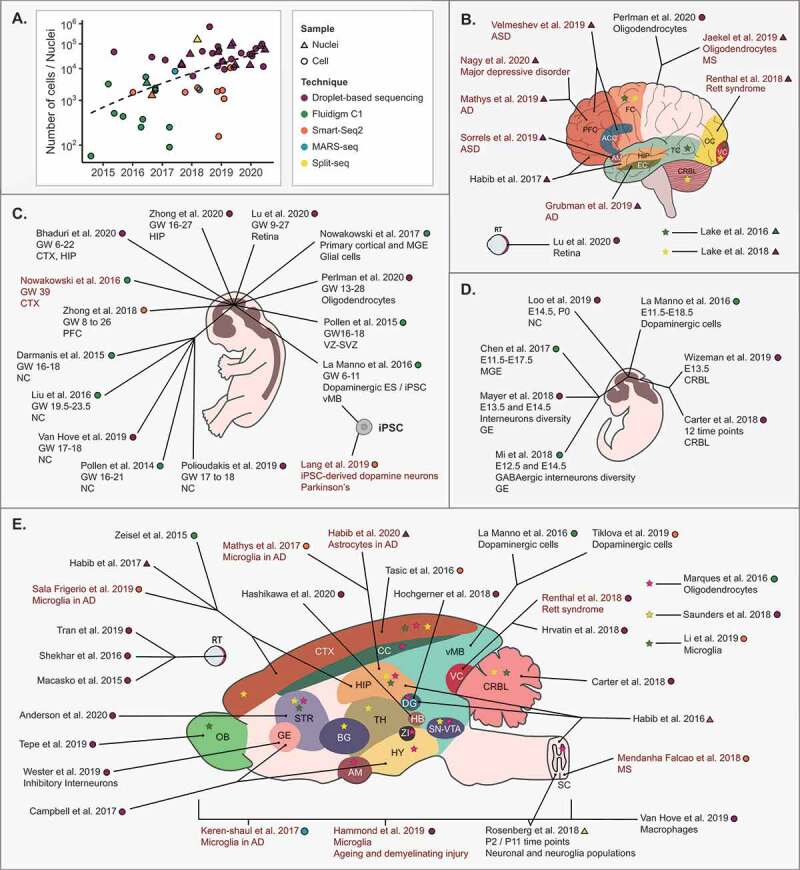
**A roadmap of scRNA-seq studies in mammalian neuroscience: A)** Progressive scaling of scRNA-seq studies in mammalian neuroscience. Individual points are coloured by the methodology used, and shaped according to whether whole cells or nuclei were profiled. **B–E)** Maps of scRNA-seq reports from the **B)** adult human brain **C)** human embryonic nervous system **D)** mouse embryonic nervous system and **E)** adult mouse brain. Abbreviations: Anterior cingulate cortex (ACC), Alzheimer’s disease (AD), Amygdala (AM), Autism spectrum disorder (ASD), Basal Ganglia (BG), Corpus callosum (CC), Cortex (CTX), Cerebellum (CRBL), Dentate Gyrus (DG), Entorhinal cortex (EC), Entopeduncular Nucleus (EP), Frontal cortex (FC), Ganglionic eminence (GE), Gestational week (GW), Habinular complex (HB), Hippocampus (HIP), Hypothalamus (HY), Induced pluripotent stem cell (iPSC), Medial ganglionic eminence (MGE), Multiple sclerosis (MS), Neocortex (NC), Olfactory bulb (OB), Postnatal (P), Prefrontal cortex (PFC), Retina (RT), Spinal cord (SC), Substantia nigra and Ventral tegmental area (SN-VTA), Striatum (STR), Subventricular zone (SVZ), Temporal cortex (TC), Thalamus (TH), Visual cortex (VC), Ventral midbrain (vMB), Ventricular Zone (VZ), Zona Incerta (ZI). Symbols follow key of part **A)** to indicate the methodology used and whether cells or nuclei were profiled. Coloured text indicates disease-relevant tissue/models were profiled

In principle, all current scRNA-seq methods involve the dissociation of a sample into single cells, then processing of the individual cells such that all the RNA derived from a given cell is demarcated with an identical barcode. Accordingly, the barcode can subsequently be used to denote the cellular origin of each sequenced transcript during downstream bioinformatics analyses. It was this introduction of the cell barcoding concept in the Single-cell Tagged Reverse-Transcription sequencing (STRT-seq) protocol [4] that initiated the method development revolution in 2011, as it moved the field from sequencing one cell at a time [5] to being able to differentiate each cell’s individual transcriptome from the next within the same dataset. As a generalization, the different protocol variants that have since emerged then differ from one another in i) their method of barcoding the RNA from individual cells (Fig. 1A), ii) their method to amplify low abundance cellular RNA (Fig. 1B), iii) the region of the mRNA transcript that is enriched and sequenced (Fig. 1C), iv) the method used to capture and process the individual cells (Fig. 1D), and v) the choice of starting material (Fig. 1E). Selected combinations of these parameters have then facilitated an exponential scaling of scRNA-seq in recent years, including in the neurosciences (Fig. 2A). Each of these is discussed in greater detail over the following sections.

### 2.1. Cellular and molecular barcoding

Demarcating a cell’s transcriptome with a unique cell barcode common to all the transcripts of that cell was initially achieved by physically isolating single cells into individual wells, lysing the cells, proceeding with individual RNA-seq library preparations that incorporated well-specific barcodes, and finally exponential amplification by PCR [4,6] (Fig. 1A, upper panel). However, this well-by-well strategy is costly and prevents early multiplexing of libraries. Accordingly, the Cell Expression by Linear amplification and sequencing (CEL-Seq) protocol [7] introduced barcode sequences into the reverse transcription primers such that cells isolated across a plate could be pooled and processed as a single multiplexed sample from an earlier stage (Fig. 1A, lower panel). This allowed 96-well plates to be processed through library preparation at a reduced cost, whilst additional plate-specific indexes introduced during PCR amplification allowed multiple experiments to be combined on a single sequencing machine flow-cell. This combination of reverse transcription-based indexing of cells and PCR-based sample indexing has since established itself in most high-throughput scRNA-seq protocols, although well-based indexing is still used in low-throughput studies where full transcript coverage is required (see section 2.2 and 2.3).

In addition to denoting a RNA’s cellular origins with a cell-specific barcode, the absolute quantification of the RNA transcripts in each cellular transcriptome is a requisite for many scRNA-seq studies. Whilst metrics used in traditional RNA-seq (e.g. FPKM, RPKM) were used early on, they are poorly suited to scRNA-seq due to the amplification and coverage biases that exist in different single-cell workflows. Introduction of unique molecular identifiers (UMIs) into the cDNA prior to library amplification provided a means to perform molecule counting by collapsing identical sequencing reads that are a consequence of amplification [8]. Initially used as an internal validation control of scRNA-seq quantification estimates [9], this has since been shown to improve the reproducibility of quantification across individual cells, particularly for low-abundant transcripts [10]. Accordingly, the use of UMIs and molecule counting has become a common feature of most scRNA-seq protocols.

### 2.2. Amplification of cellular transcriptomes

The low abundance RNA in individual cells currently necessitates an amplification step prior to NGS (Fig. 1B). Following the early studies of Iscove and Eberwine, both linear amplification with IVT and exponential amplification with PCR have been incorporated into more recent protocol adaptations. IVT has the advantage that linear amplification better preserves relative abundances of mRNA transcripts whilst avoiding exponential amplification of any errors or the biases that exist in other methods towards certain high-abundance transcripts. In such techniques, it is the reverse transcription primer that additionally introduces the T7 polymerase promoter required for IVT amplification [7]. However, successive rounds of amplification each lead to progressive transcript shortening that manifests as a 3ʹ end enrichment [11]. The pooling of barcoded cells in the Massively Parallel single-cell RNA-Sequencing (MARS-seq) [12] and CEL-seq [7] protocols has increased starting input and mitigates this bias through reduced rounds of amplification, but the labour-intensive nature means their uptake has been limited.

PCR provides a readily implemented alternative for most labs. It can be achieved following a template-switching reverse transcription reaction to incorporate a sequence-specified PCR handle at the 5ʹ end of cDNA derived from oligo-dT primed mRNA transcripts (Fig. 1A, lower panel). First introduced in Clontech’s Switching Mechanism at 5ʹ End of RNA Template (SMART) protocol for amplification of low-abundant RNA, this provides the necessary primer sequences at both ends to allow full-length cDNA amplification (Fig. 1B). The method has since been heavily optimized for NGS in the SMART-seq [13], SMART-seq2 [14] and SMART-seq3 [15] workflows, and we herein refer to all three adaptations simply as ‘SMART-seq’ unless specified otherwise. Various protocols subsequently differ in their use of the full-length cDNA. Well- or chamber-based scRNA-seq workflows, such as SMART-seq and Fluidigm C1, fragment the cDNA before adding the previously described well-specific indexes during PCR. Accordingly, each cDNA fragment is cell indexed and full length scRNA-seq can be achieved. Higher throughput scRNA-seq adaptions alternatively incorporate non-cell specific adapters, typically by tagmentation, that are used with termini-specific primer sequences to amplify either the 3ʹ or 5ʹ regions of mRNA transcripts. Notably, these amplified regions additionally contain the cellular barcodes that have instead been introduced during reverse transcription. As described in the previous section, the early introduction of cell barcodes during reverse transcription enables early multiplexing prior to any amplification that can facilitate increases in scale. However, despite the flexibility, PCR-based methods have the disadvantage that the required template-switching has low efficiency and is influenced by mRNA sequence and the presence of a 5ʹ-m^7^G cap [16]. Meanwhile, both highly expressed transcripts and shorter cDNAs are biased during the exponential amplification step. Recent reports have shown template-switching efficiency can be improved through various subtle changes to the reaction conditions [15,17], whilst the Quartz-seq2 method replaces template-switching with a terminal transferase reaction that introduces a poly-A tail to the cDNA [18]. This is then utilized in second-strand synthesis to introduce a PCR handle for full-length cDNA amplification. Both developments increase cDNA library complexity, so it is expected they could be integrated into existing methods in the near future.

Isothermic rolling circle amplification of circularized cDNA with *phi29* polymerase provides an additional alternative that is able to preserve full-length transcript coverage and relative abundances from single cells (Fig. 1B) [19]. Integration into scRNA-seq protocols has been limited to date, although it has recently provided opportunities for full length transcript sequencing with emerging nanopore technology [20]. It will be interesting to see whether this amplification method can be integrated into existing protocols in future years, or alternatively, whether direct RNA sequencing with nanopore technology can eliminate such amplification steps altogether.

### 2.3. Transcript coverage

As noted in the previous section, different PCR-based workflows result in enrichment of different parts of an mRNA transcript. Specifically, and following full-length cDNA production, either the full mRNA transcripts can be processed and sequenced, or the 5ʹ/3ʹ regions of an mRNA can be enriched (Fig. 1C). Full-length coverage is presently only possible in a relatively low-throughput manner due to the challenges in providing all fragments from the same transcriptome with the same cellular barcode, and the increased sequencing requirements to cover a cell’s full exome that may become cost-prohibitive as cell numbers increase. In contrast, capturing a smaller part of the exome enables an increased level of cell multiplexing within the current confines of sequencing capabilities to facilitate higher throughput studies. However, whilst increased scale remains tempting to many, the choice of the protocol should carefully consider the question in mind.

After UMIs are factored in, methods enriching transcript ends should in theory provide just a single cDNA per RNA molecule rather than the many scattered cDNAs across a transcript seen in full-length workflows. Mis-priming during reverse transcription on internal poly-A sequences means this is not always the case, although these can be computationally identified and filtered out [21]. The enrichment of termini in conjunction with appropriately designed UMIs can thus provide robust quantification of gene abundance that is comparatively immune to gene length biases evident in full-length methods. Accordingly, these workflows are well suited for summarizing differential gene expression changes across cells. This can be leveraged for granular dissection of cell heterogeneity [22–24], paradigm dependent changes in gene expression profiles of specific cell populations [25], or reverse-engineering underlying gene regulatory networks with systems biology workflows [26,27]. Termini-specific features can also be monitored across individual cells. For example, 5ʹ enrichments have been leveraged for immune profiling of B or T cells [28], whilst 3ʹ enrichments have been used to identify cell-specific alternative poly-adenylation patterns [29]. Due to biases mentioned in this and previous sections, such analyses tend to only accurately report upon the most highly expressed genes. However, novel computational methods that share data across cell populations can provide enhanced opportunities to make accurate calls on lower expressed transcripts [30,31].

Whilst the lower throughput full-length cDNA workflows also permit the above, they have also impressively enabled single-cell analysis of isoform usage and pre-mRNA splicing decisions in certain studies [9,32,33]. It should be noted that the ability to accurately detect such changes is complicated by typical low depths of the sequencing of individual cells, technical noise introduced by amplification steps, variable mRNA capture efficiencies, and technical limitations imposed by different sequencing technologies. Indeed, whilst tools for detecting alternative pre-mRNA processing in bulk RNA-seq generated with short-read sequencing have shown promise on simulated single-cell datasets [34], studies utilizing experimental short-read datasets have required sophisticated algorithms that factor in the associated technical noise and learn to read distributions to detect [32,35] and quantify [33] pre-mRNA processing changes. Long-read sequencing offered by Oxford Nanopore Technologies (ONT) and PacBio provides an attractive alternative solution to these challenges, and has already identified new isoforms that are absent from the latest annotation databases [36]. Accordingly, there could be much potential for applying long-read sequencing to study pre-mRNA processing in the scRNA-seq field. However, current ONT-based studies have reported error rates that preclude accurate transcript quantification using UMIs [20], whilst the more accurate but lower throughput PacBio platform has required increasing depths of sequencing to ensure analysis extends beyond a limited number of genes [36,37]. In future, it will therefore be exciting to see whether integrating error-corrected nanopore sequencing into scalable scRNA-seq workflows presents alternative opportunities for accurately studying isoform-level expression patterns at cell resolution [38,39].

### 2.4. Scaling up

Despite identifying methods to barcode and separate individual cell transcriptomes from one another in multiplexed sequencing reactions, manual isolation of single cells into individual wells restricted initial studies to the profiling of relatively few cells (~10^2^, Fig. 1D upper panel). This could be modestly increased with automated FACS-based sorting of cells [12] and integrated microfluidic circuits [6] to ~10^2^–10^3^ cells per the study, but a major challenge to the field was to scale up and profile increasing numbers of cells whilst maintaining quality and integrity. Three elegant solutions have since been identified to overcome this problem, each utilizing different high-throughput capture methods that retain the ability to barcode cells prior to multiplexed library preparations (Fig. 1D, lower panel).

The most simplistic in principle has been to increase the number of wells being used to capture and barcode cells. To achieve this, Bose et al. initially used polydimethylsiloxane (PDMS)-based printing of >10,000 pico-wells (50 μm in diameter) on a solid surface to facilitate the gravitational capture of ~100 individual cells in every 1000 wells after being introduced across the surface in a single-cell suspension of limited dilution (Fig. 1D, lower left panel) [40]. Co-capture of a uniquely barcoded mRNA capture bead in the well by the same gravitational method then subsequently introduced oligos required for cell barcoding and library preparation. Specifically, each individual bead is coated with oligos containing a unique bead-specific barcode, oligo(dT), and sequences required for library amplification. On-chip lysis, reverse transcription and amplification could then be carried out before indexed cell transcriptomes can be multiplexed and processed for sequencing. Subsequent variants of this scaling principle have now reduced the need for custom chip processing equipment, and improved cell separation by using semi-permeable membranes that help reduce well-to-well contaminations [7,41,42]. Meanwhile, the cost-effective workflow has been used to collect and profile >10^4^ cell transcriptomes in some studies.

The second solution involved the development of microfluidic technology to co-capture single cells in individual aqueous droplets in oil together with aforementioned uniquely barcoded mRNA capture beads (Fig. 1D, lower middle panel) [22,43]. Incorporation of lysis buffer during droplet formation subsequently leads to the release of the cellular RNA and its capture on the beads in order to demarcate transcripts with a cell barcode. Two main flavours of the workflow then exist: reverse transcription within droplets followed by droplet bursting, bead pooling and multiplexed library preparation [43,44], or droplet bursting and bead pooling immediately after RNA capture ahead of a single multiplexed reverse transcription reaction and library processing [22,45]. The first has the advantage that deformable beads are used that permit high droplet occupancies (~80%), whilst the second remains more frugal on reagent costs. Commercial platforms based on both methods now allow 8 samples to be processed in parallel, with ~5-8 × 10^4^ transcriptomes captured from each sample in ~15 minutes [44,45]. So-called droplet-based sequencing has thus become a popular method across the life-sciences due to its scalability and availability of multiple commercial platforms facilitating the approach [43–45]. Indeed, >1 × 10^6^ neural cells have been profiled in one such dataset generated by 10x Genomics Ltd for the unrestricted use of the research community [31].

Despite their simple-to-implement workflows, both aforementioned strategies have the disadvantage of needing specific lab equipment (e.g. FACS, custom fabricated chips, droplet generator, barcoded mRNA capture beads). This makes these techniques expensive, technically challenging and inaccessible for many groups. A third strategy addresses these obstacles by using the cells themselves as reaction chambers for *in situ* mRNA barcoding. Specifically, the sci-RNA-seq [46] and SPLiT-seq [24] protocols begin by introducing barcoded reverse transcription primers and reverse transcription reagents into semi-fixed and permeabilized cells in a standard plate-based format (Fig. 1D, lower right panel). Notably, pools of cells are provided to each well such that many cells will have the same initial barcode. Subsequent rounds of cell pooling followed by redistribution across reaction plates and further barcode additions by either *in situ* ligation or PCR amplifications then ensures each individual cell follows a different path through the protocol to build a unique barcode relative to its initial neighbours. Increasing the number of barcoding steps increases barcode diversity and scaling potential whilst reducing the probability of two cells returning the same barcode. This has so far been restricted to four steps in SPLiT-seq where >1.5 × 10^5^ cells from the mouse brain and spinal cord were profiled in a single experiment using no customized equipment [24].

Whilst these represent the main strategies for scaling scRNA-seq at present, the recent combination of the last two concepts has excitingly opened up new opportunities for ultra-high throughput scaling in future using the ‘single-cell combinatorial fluidic indexing’ (scifi) RNA-seq workflow [47]. Here, pre-indexing of mildly fixed cells with an *in situ* reverse transcription reaction subsequently allows multiple cells to be loaded into individual oil droplets using already mature microfluidic technology. This mitigates the requirement for precise loading densities of cells onto the microfluidic devices to restrict co-capture of multiple cells in a droplet with an mRNA capture-bead. More importantly, it enables a 15-fold increase in the throughput of droplet-based scRNA-seq and provides a simple strategy for multiplexing hundreds of samples in a single experiment.

### 2.5. Choice of starting material

Where feasible, scRNA-seq has been carried out on viable, whole cells isolated from cultures or tissue (Fig. 1E). Such preparations can be made using enzymatic separation of complex samples [48–50], manual straining of samples through micro-metre meshes [51], or combinations of both [52–54]. However, the requirement for whole cells can be limiting to research requiring clinical samples, archived tissues, tissues sensitive to enzymatic dissociation, or tissues with an architectural complexity that is non-trivial to dissociate into intact single-cell suspension. Neural tissue presents a particularly difficult challenge because of its cellular complexity. Developing brains have lower connectivity as the neural network is not yet developed, and this can allow dissociation of embryonic samples into single-cell suspensions [48–54]. However, neural cells in adult brains form long axons and numerous connections to other cells such that high integrity dissociation into intact cells is nigh on impossible. Indeed, certain motor and sensory neurons in the human CNS send out projections >1 m in length that are impossible to fully dissociate from their surrounding cells and environment.

To address this challenge, single-nuclei RNA-seq (snRNA-seq) has emerged as a viable alternative option. Of crucial importance here, single nuclei can be collected in high quantity and quality from such samples with comparatively gentler methods than the often-harsh enzymatic digestions used in scRNA-seq. This includes from archived frozen tissue, although it is important to appreciate that processing such frozen samples can result in variable thawing speeds and RNA quality between the cells or nuclei in the periphery and those in the centre. Controlled thawing of samples should therefore be carefully optimized to avoid poor dataset metrics, and it is thus noteworthy that current snRNA-seq studies from the adult human brain have prepared nuclei from cryostat sectioned tissue where thawing speeds across samples are expected to be more even than from a thicker tissue block [55]. A number of snRNA-seq protocol variants have now been reported that follow the developments made in scRNA-seq. Indeed, well-based variants [56–58] have since been followed by droplet-based [23,57,59] and *in situ* based workflows [24] to permit increased scalability. Crucially, several studies have now directly compared scRNA-seq and snRNA-seq from identical samples. In addition to confirming expected findings such as increased intronic and reduced mitochondrial RNA alignments in snRNA-seq, these studies have consistently reported good agreement between gene-expression signatures of isolated cells and isolated nuclei [23,57,60–62]. As will be seen in the following sections, this has subsequently enabled snRNA-seq to report granular characterizations of the myriad different cell types in challenging-to-study tissues such as the human CNS [23,24,57].

Excitingly, the development of snRNA-seq presents an unprecedented opportunity to access human tissue banks in a fundamentally new way in order to enhance our cellular understanding of disease directly in the context of the human condition. This is extremely important for the study of neurological disease given the species differences that exist between humans and other model systems. For example, human neuroglia have a ~10-fold increase in processes, a ~25-fold increase in volume, and support ~20-fold more synapses than rodent neuroglia. Meanwhile, the frontotemporal lobar degeneration (FTLD)-vulnerable large spindle neurons of the anterior cingulate cortex are primate-specific [63]. Similarly, the amyotrophic lateral sclerosis (ALS)-vulnerable gigantopyramidal layer V Betz cells are not detected in rodents, whilst they project further, have larger somas and have dramatically thicker axons than rodent corticospinal motor neuron counterparts [64]. Accordingly, certain human neuropathologies will never be fully reproduced in rodents. The potential for snRNA-seq to further dissect the cellular dynamics of human-diseased tissue is thus of huge importance if we are to truly understand the human condition.

### 2.6. Single-cell multi-omics

A common complement in traditional transcriptomics study has been assessing the relationship to other measurements from the sample such as epigenomic or proteomic signatures. Such multi-modal information can provide more holistic views of a given sample. It therefore comes as no surprise that the technological developments in the field have allowed several groups to start implementing single-cell multi-omic workflows. A full discussion on all single-cell multi-omic methods is beyond the scope of this review, and instead, we redirect the reader to an excellent recent review on the topic [65]. We instead highlight two concepts of multi-omic studies with examples; parallel processing of modalities and combined processing of modalities.

Genome and Transcriptome (G&T)-seq employs parallel processing of modalities, and is designed for integrative analysis of genomes and transcriptomes from the same single cell [66]. This is achieved by an early biotin-based separation of cell transcriptomes from the cellular DNA following the reverse transcription step of a modified SMART-seq protocol. Parallel processing of the DNA and the RNA libraries are then carried out through different workflows and separated sequencing. The method can be used to identify causative genetic variations that relate to variable cell-to-cell expression. An advantage of parallel processing in this setting is that overlapping genomic and transcriptomic reads at exons are separated at an early stage, thus allowing accurate calling of copy number from the genomic DNA without a non-trivial *in silico* filtering of mRNA reads.

The alternative is to keep the modalities together and use a common strategy to capture both. This can be more convenient, reduce the opportunity for contamination, and limit opportunities for material loss. For example, Cellular Indexing of Transcriptomes and Epitopes (CITE)-seq allows characterization of the cell surface proteome and transcriptome of the same single cell [67]. To achieve this, protein-specific barcode oligonucleotides are conjugated to antibodies that target desired cell surface proteins. The tagged antibodies are next incubated with cell suspensions prior to scRNA-seq, with the tagged antibodies carried into the cell isolation chamber (e.g. an oil droplet) if the protein-of-interest is detected. Since the conjugated oligonucleotides additionally contain a poly-A tail such that they mimic an mRNA, protein detection can subsequently be reported in the resulting datasets due to the capture of the barcode oligonucleotide on the mRNA capture beads alongside other cellular transcripts. This method has shown excellent agreement with FACS-based study and is convenient to implement. Like with the choice of scRNA-seq workflow then, the parallel or combined processing of modalities in multi-omic studies must thus be carefully considered for the desired application.

### 2.7 . Bioinformatic challenges

Independent of the technique chosen or biological question investigated, all scRNA-seq experiments will generate large sequencing datasets requiring bioinformatics analysis. Analysis workflows typically include the same two initial steps: quality control and pre-processing of the raw data (i.e. demultiplexing, genome alignment, normalization, data correction, feature selection, dimensionality reduction). Several tools developed in other fields are applicable for each step but the integration of many independently developed tools into a scRNA-seq compatible workflow can be nontrivial for those entering the field. Accordingly, several excellent and easy-to-implement scRNA-seq workflows have been developed that now allow their ready integration. Among others this includes Drop-seq tools [22], DropSeqPipe [68], Cell Ranger [44] and Seurat [69]. Either individually or in combination, such workflows can take the user from raw data to individual cell transcriptomes that have been clustered according to their expression of marker genes. Moreover, recent refinements to some of these well-documented packages can overcome additional challenges such as aligning data from different replicates and technologies, profiling increased cell numbers, accounting for the level of ‘noise’ derived from different scRNA-seq protocols, the biological heterogeneity of cells and batch effects.

Downstream analyses of pre-processed scRNA-seq datasets may then be cell- or gene-level. Cell-level analysis may include more granular cell clustering [23,24] and trajectory inference methods [70,71], while gene-level analysis can include differential gene expression [72] and gene regulatory network inference methods [26]. Here researchers need to carefully choose the most appropriate tool for their dataset and biological question. In this regard, we refer the reader to recent reviews that expand on best practices of data pre-processing and downstream analysis [73,74].

## 3. Lessons learnt from single-cell transcriptomics in the mammalian CNS

The mammalian CNS is an extremely complex network of highly differentiated cell types that develop through highly specialized steps (e.g. migration, neuritogenesis, axon guidance) and have unique and specific functions, which are, in some way, compromised in disease. The use of scRNA-seq or snRNA-seq on CNS tissue has provided new opportunities to identify new cell types, disease-specific cell states, identify cell-type-specific molecular changes, study disease progression and dissect molecular mechanisms with high-resolution. In the following sections we briefly discuss how scRNA-seq has dramatically expanded our knowledge of cell diversity in the mammalian CNS, then review what it has taught us about various neurodevelopmental and neurodegenerative disorders. To see how scRNA-seq has informed us about mechanisms of CNS cell specification during neurodevelopment, we refer the reader to a complementary recent review [75].

### 3.1. Cell diversity of the nervous system

As with scRNA-seq studies across the life sciences, application of scRNA-seq to the mammalian CNS has heralded a comprehensive characterization of both new and existing cell populations. In mice this has included the profiling of whole embryonic brains and spinal cords, and the more focussed study of many major brain regions in both mice and humans. Other studies have enriched specific cardinal cell populations to obtain more granular separations within these. Among others, dopamine neurons [76], microglia [77] and retinal cells [22,78] have all been enriched for population-specific studies due to their important biological and disease-relevant roles. A full discussion of each brain region’s cell-type diversity is beyond the scope of this review, and we instead provide a roadmap to those interested in specific brain regions or developmental time-points (Fig. 2, Table 2). Taken together though, these studies have overwhelmingly emphasized the incredible diversity of cell populations found across the mammalian CNS. It remains unclear precisely how many new cell types remain to be discovered, but their characterization now provides an unprecedented platform to both understand human health and neurological disease. Looking forward, it will now be a formidable challenge to understand which cell populations are common across studies, and to assemble a comprehensive reference map of the human brain as part of the international human cell atlas [79].

**Table 2.** **Murine and human CNS cell clusters inferred with scRNA-seq or snRNA-seq. Studies listed are those portrayed in** Fig. 2

Species	Region/Cell type	Cell clusters	Reference
Mouse	Olfactory bulb	38	^[134]^
Mouse	Medial ganglionic eminence	12	^[48]^
Mouse	Ganglionic eminence	14	^[135]^
Mouse	Caudal ganglionic eminence, primary visual cortex	15	^[136]^
Mouse	Subpallium, dorsal and ventral medial ganglionic eminence, caudal ganglionic eminence	48	^[52]^
Mouse	Hippocampus, thalamus, posterior cortex, cerebellum, substantia nigra and ventral tegmental area, Entopenduncular and subthalamic nuclei, Globus pallidus and nucleus basalis, striatum, frontal cortex	565	^[51]^
Mouse	Arcuate hypothalamus, median eminence	50	^[137]^
Mouse	Spinal cord, whole brain	113	^[24]^
Mouse	Ventral midbrain (embryonic), sorted adult dopaminergic neurons	31	^[53]^
Mouse	Cerebral cortex	44	^[49]^
Mouse	Cerebellum	19	^[54]^
Mouse	Cerebellum	48	^[50]^
Mouse	Somatosensory cortex and hippocampal CA1 region	47	^[138]^
Mouse	Primary visual cortex	49	^[139]^
Mouse	Primary visual cortex	30	^[100]^
Mouse	Primary visual cortex	8	^[92]^
Mouse	Hippocampus, prefrontal cortex (adult)	21	^[23]^
Mouse	Retina	46	^[99]^
Mouse	Retina	39	^[22]^
Mouse	Cortex and hippocampus	47	^[138]^
Mouse	Habenula complex	20	^[101]^
Mouse	Striatum	43	^[140]^
Mouse	Hippocampus, dentate gyrus, spinal cord	29	^[58]^
Mouse	Dentate Gyrus	22	^[141]^
Human	prefrontal cortex, motor cortex, parietal cortex, somatosensory cortex, primary visual cortex, hippocampus (foetal)	42	^[142]^
Human	Ventral midbrain (embryos)	25	^[53]^
Human	Neocortex, neural precursor cells	4	^[6]^
Human	Neocortex	7	^[95]^
Human	Ventricular zone, subventricular zone (embryos)	16	^[96]^
Human	Ventricular zone, subventricular zone, subplate, cortical plate (embryos)	16	^[86]^
Human	Neocortex	7	^[143]^
Human	Prefrontal cortex (embryos)	35	^[144]^
Human	Hippocampus (embryos)	47	^[145]^
Human	Neocortex, medial ganglionic eminence	11	^[146]^
Human	Hippocampus, prefrontal cortex	15	^[23]^
Human	Amygdala	15	^[147]^
Human	Prefrontal cortex	26	^[148]^
Human	Frontal cortex, temporal cortex, visual cortex	17	^[133]^
Human	Visual cortex, frontal cortex, cerebellum	35	^[57]^
Human	Retina	126	^[149]^
Human	Occipital cortex	9	^[92]^
Human	Prefrontal cortex, anterior cingulate cortex	17	^[55]^
Human	White matter	6	^[150]^
Mouse	Dopamine cells of ventral midbrain	7	^[76]^
Mouse	Microglia across ageing	9	^[151]^
Mouse	Microglia of cortex, cerebellum, hippocampus, striatum, olfactory bulb, and choroid plexus	15	^[152]^
Mouse	Whole brain and border region macrophages	15	^[153]^
Mouse	Retinal bipolar cells	26	^[78]^
Mouse	Oligodendrocytes of Somatosensory cortex, striatum, dentate gyrus, hippocampus CA1, Corpus callosum, amygdala, hypothalamus, zona incerta, SN-VTA, dorsal horn	13	^[154]^

### 3.2. Neurodevelopmental disorders

Beyond characterizing cell-type diversity of the CNS in healthy conditions, scRNA-seq has enabled new insights into various disorders and diseases of the nervous system. In the case of neurodevelopmental disorders, several recent reports have emerged tackling specific conditions. We accordingly consider what scRNA-seq has taught us so far about Autism Spectrum Disorders (ASDs), Rett syndrome, Zika Virus infection and generalized seizure activity.

#### 3.2.1. Autism Spectrum Disorder

ASD is an umbrella term for closely related neurodevelopmental conditions that impact how a person perceives and socializes with others, causing problems in social interaction, repetitive behaviours and communication. Whilst much progress has recently been made characterizing the convergent transcriptomic mechanisms and signatures which are characteristic of ASDs at the bulk RNA-seq level [80,81], these studies have been limited in their ability to determine the precise cell types affected with high resolution. In an attempt to address this, snRNA-seq of the healthy adult neocortex has recently been used to identify those cell-types that are enriched in the differential gene expression and alternative splicing programmes that are consistently detected by bulk RNA-seq of ASD tissue [55,82]. Specifically, snRNA-seq identified endothelial cells, microglia and astrocytes to be most highly enriched for a set of 27 genes related to immune system response that are consistently differentially expressed across four cortical (frontal and temporal) regions in bulk RNA-seq. Activation of these glial cell populations is consistent with the previously documented involvement of both astrocytes and microglia in the neuroinflammation present in ASD pathology [83–85]. Further to this, snRNA-seq dataset implicated projection neurons and interneurons as being enriched for ASD-relevant alternative splicing signatures identified in bulk RNA-seq in healthy adult cortex, with the genes involved predominantly linked to synaptic function. However, whilst the study goes some way to identify cortical cell types implicated in ASD, the comparison to adult human cortex may not accurately account for developmental-stage specific gene expression programmes.

A recent scRNA-seq atlas of human neocortex at a period implicated in the onset of various neurodevelopmental disorders [86] (gestation weeks 17–18) has provided a pertinent alternative for this gene enrichment-style approach. Indeed, this atlas has been used to evaluate cell-type-specific expression of genes related to neurodevelopmental disorders at this critical juncture, such as genes genetically associated with ASD [87]. This revealed that the majority of high-confidence ASD-risk genes are enriched in developing excitatory glutamatergic neurons within deep and upper cortical layers, although the precise distribution of the individual genes varies: some display pan-neuronal expression (e.g. *MYT1L, AKAP9*), others cell subtype-specific expression (e.g. *GRIN2B*), whilst some are specific to progenitor populations (e.g. *ILF2*). Meanwhile, certain ASD-risk genes also display distinctly non-neuronal expression (e.g. *TRIO* and *SETD5* in oligodendrocyte precursor cells, *SLC6A1* in pericytes) to imply a still poorly understood contribution of neuroglia and the blood-brain barrier to ASD aetiology [86]. Beyond ASD, expression risk genes implicated in both epilepsy and intellectual disability (ID) were found to be generally enriched in sub-types of glutamatergic neurons, whilst certain high-risk ID genes are highly expressed in radial glia, a distribution not observed for epilepsy or ASD-risk gene sets. Thus, whilst partial overlap exists in the genes implicated in each of these disorders, the individual cell-type-specific expression patterns of each gene set are unique.

Whilst using healthy brain tissue can aid in the identification of cell-types likely affected in ASD, it is limited in providing direct information regarding the disease state. Assessment of ASD post-mortem pre-frontal (PFC) and anterior cingulate cortex via droplet-based snRNA-seq have therefore provided a complementary context-specific insight into the key cell types affected, and their organization within the cortex [55]. Specifically, to dissect primary effects of ASD from potential secondary gene signatures associated with seizure activity, a common co-morbidity with ASD, the cohort consisted of 15 ASD post-mortem cases between 4 and 22 years of age, with age-matched controls, and a further assessment of the PFC from patients with epilepsy. Interrogation of the composition of the ASD tissue revealed an increase in the proportion of both protoplasmic astrocytes and layer IV excitatory neurons in one of the two cortical regions assessed. Additionally, specific cell types in the ASD tissues displayed differential gene expression profiles relative to controls. This particularly included the downregulation of neuronal genes in cortical excitatory neurons of layer II/III and layer IV, including genes linked to synaptic function (e.g. *STX1A, SYN2, NRXN1*) and neurodevelopment-associated transcription factors (e.g. *TCF25, SOX5, RBFOX3*), and to a lesser extent, in VIP-expressing inhibitory interneurons (*RAB3A, TCF25, AHI1*). The most affected gene ontology (GO) terms were related to chemical synaptic transmission, axon guidance, neuronal migration and GABA signalling. Concurrently, microglia displayed the highest proportion of positive differentially expressed genes (DEGs) and were enriched for genes linked to microglial activation and transcription factors affecting development. Again fitting with the documented neuroinflammation present in post-mortem ASD brains [83,84], protoplasmic astrocytes displayed upregulation of transcription factors affecting development (e.g. *TCF25, LMO3, SOX5*) and cell mobility (e.g. *FIGN, DLC1, CADM2*) alongside their increased abundance.

Interestingly, specific subsets of DEGs within layer II/III neurons and microglia could be correlated to the severity of the presenting ASD phenotype. Further to this, cell-type-specific DEGs were over-represented by those considered to confer high-confidence ASD risk, such as those listed by the SFARI database as genetically associated ASD risk genes [87]. The overlap in cell-type-specific changes was low between the ASD and epilepsy group. The greatest affected DEGs in the epilepsy cases presented in layer V/VI corticofugal projection neurons and parvalbumin interneurons. This led to the conclusion that the majority of the cell-type-specific DEGs observed in ASD were primary to the disease, and not a result of linked epilepsy.

#### 3.2.2. Rett syndrome

Rett syndrome is a X-linked neurodevelopmental disorder that primarily affects females and is characterized by autistic-like features. It results from a mutation of *MECP2* located on the X-chromosome. *MECP2* encodes for a neuron-enriched protein which binds to methylated cytosines and which may subsequently act as a transcriptional repressor of methylated genes. Other implicated functions include mRNA splicing, transcriptional activation and chromatin remodelling [88–91]. Due to random X-chromosome inactivation in females, carrying a single defective *MECP2* allele can lead to mosaicity in the brain, whereby there is coexistence of cells expressing either wildtype or mutant MECP2 protein within affected heterozygous individuals. Cell-type specific gene expression has recently been determined of post-mortem occipital cortex from human Rett syndrome patients via snRNA-seq, as well as from the visual cortex of *Mecp2*^+/-^ mice that phenocopy features of Rett syndrome [92]. By correlating single-nucleotide polymorphisms (SNPs) expressed in *cis* to *MECP2* that are distinct for either the wild-type or mutant allele, faithful assignment of the transcriptome of each mosaic cell was achieved. Accordingly, this overcomes the inability to directly sequence the relevant presenting *MECP2* gene mutations with the 3ʹ-biased snRNA-seq approach used. This analytical technique, termed ‘SNP-seq’, allowed for the interrogation of both cell-autonomous and non-cell autonomous transcriptomic signatures which contribute to Rett syndrome pathophysiology within individual cases. This was able to recapitulate previous findings of cell-autonomous effects of *MECP2* loss [93,94], with an observed upregulation of highly methylated long genes in excitatory neurons confined to those cells expressing the mutant MeCP2 protein in both the patient and mouse cortex samples. Through comparison to solely wild-type mice, non-cell autonomous signatures within wild-type excitatory neurons in **Mecp2*^+/-^* mice were also discerned. This revealed enrichment for DEGs involved in neuronal activity-dependent gene expression (e.g. *Brinp, Nptx2*). Interestingly, an overlap of a set of cell-autonomous DEGs was observed in mutant excitatory neurons in both the *Mecp2*^+/-^ mouse model and the human post-mortem cases (58 upregulated, 84 downregulated). The evolutionarily conserved and upregulated DEGs displayed enrichment for gene-body methylation, potentially identifying these genes as the elusive direct targets for MeCP2-mediated repression. This gene set was enriched for terms related to neuronal gene expression and for genes which have been shown to be mutated in ID and ASD, including transcriptional regulators *AUTS2* and *RBFOX1*.

Looking forward, whilst the approach was applied solely to *Mecp2* deficient adult mice, this approach could now be extended to assess the cell-specific aspects of Rett syndrome progression throughout gestational neurodevelopment. Meanwhile, the SNP-seq approach could also allow for the study of further X–inactivation-linked NDDs, including Fragile-X syndrome, *CDKL5* deficiency disorder or X-linked intellectual disabilities.

#### 3.2.3. Zika virus

Zika virus (ZIKV) impacts neurodevelopment to cause foetal abnormality and microcephaly following maternal infection. This particular susceptibility of the developing brain raises the important question of whether key molecular features of neuronal progenitor cells make the developing brain uniquely susceptible to ZIKV compared to adult tissue. To address this, a scRNA-seq dataset from human cortex at gestational weeks 16–18 was used to elucidate the cell-type-specific expression patterns of receptors implicated in the entry of certain viruses known to cause neurodevelopmental phenotypes [95]. Whilst several candidates that could potentially facilitate viral entry to cells were expressed across the developing cortex, the gene encoding the AXL receptor stood out as displaying enriched expression within radial glia; a progenitor population considered to act as neural stem cells. Notably, this included expression within recently identified outer radial glial populations [96], whilst *AXL* was additionally present in the radial glia-like and outer radial glia-like populations of human iPSC derived cerebral organoids [95]. However, immunohistochemistry crucially confirmed AXL was excluded from mature neuronal populations at later stages of development and in the cerebral organoids. Lending further support for ZIKV entry via neuronal progenitor cells and not mature neurons, high expression of *AXL* also presented in progenitor cells of the developing retina. Accordingly, this aligns with the high concurrence of microcephaly and ocular abnormalities, whilst the unique expression of *AXL* within radial glial populations offers an explanation to the severe neurodevelopmental prenatal phenotype upon ZIKV infection.

Whilst this scRNA-seq study provided a tantalizing entry mechanism for this devastating virus, it should be noted that a later study found AXL was not required for ZIKV infection of cerebral organoids. Indeed, AXL knockout did not ameliorate the loss in organoid mass upon infection [97]. Whilst this might throw into question whether AXL is indeed required for the destructive effects of ZIKV infection, a growing body of data continues to implicate AXL in the assistance of ZIKV viral entry and modulation of the downstream immune response in infected cells. However, the cell types which are now most implicated are astrocytes and microglia [98], not radial glia. It is then noteworthy that these other cells were also found to highly express *AXL* in the scRNA-seq study [95]. Meanwhile, it continues to raise hope that inhibiting AXL function may represent a potential target for future antiviral therapies.

#### 3.2.4. Seizures

Advances in scRNA-seq workflows raise the possibility of assessing acute gene expression changes incurred in response to external stimuli at a single-cell resolution. The expression of immediate-early genes (IEGs) is rapidly and transiently induced upon various external stimuli, and is often used as a sensitive marker for activation of neural cell populations. However, aberrant induction of IEGs may also occur during tissue preparations stages of scRNA-seq workflows, which could interfere with the monitoring of treatment-specific gene induction. A recent adaptation to conventional tissue dissociation methods, incorporating the use of the general transcription inhibitor actinomycin D and lower preparation temperatures, was found to minimize artificially induced IEG expression [25]. This approach, termed ‘Act-seq,’ allowed for more accurate assessment of baseline single-cell transcriptomes and sensitive detection of treatment-specific gene expression changes. The sensitivity that Act-seq confers allowed for the assessment of activation of a range of IEGs upon chemically induced seizures in the mouse medial amygdala (MeA). Seizure-specific IEG induction programmes across different cell types were identified for neurons, astrocytes, microglia, oligodendrocyte precursor cells, endothelial cells and mural cells, all of which would have been otherwise masked by aberrant baseline IEG induction. Crucially, only a subset of each cell type displayed IEG induction upon activation. Indeed, whilst a curated list of 139 IEG genes were monitored, cell-type-specific induction of each individual gene was identified. For example, microglia, endothelial cells and mural cells displayed the highest induction of *Dusp1, Nr4a1* and *Fos*, whilst *Cyr1* was highly induced solely in mural cells (i.e. pericytes and vascular smooth muscle cells). Further testing of the Act-seq adaptation allowed for subtle neuronal-subpopulation specific IEG induction to be identified upon application of a milder immobilization stress. Distinct subpopulations of MeA neurons activated with this milder treatment, including two *Cck*-expressing subpopulations displaying the highest induction of IEGs.

The Act-seq approach is increasingly incorporated into studies where precise measurements of acute gene expression in response to physiological stimuli are required [99–101]. The findings also have implications for all studies where scRNA-seq is to be performed on freshly dissociated brain samples, as IEG induction in response to conventional dissociation was, to some extent, found to interfere with intra-cell-type clustering methods [25]. Whilst the initial application of Act-seq has focused on the cell-specific effects of both acute stress and chemically induced seizures on activity-dependent expression, potentially mimicking the effects of epilepsy, this approach could be utilized in future in other neurodevelopmental disorders which have activity-dependent aspects of disease contribution. For instance, many high-risk genes previously implicated in ASDs have roles within activity-dependent signalling pathways that relate to synaptic function [102]. Applying Act-seq to assess activity-dependent changes to transcriptomic signatures upon neuronal excitation in animal models of ASDs, including response to environmental stimuli, may aid in the identification of gene expression induction which may be perturbed in these cases, and the specific cell lines which mediate such acute responses.

### 3.3. Neurodegeneration

As with neurodevelopmental disorders, several studies have similarly used scRNA-seq to investigate the cellular and molecular mechanisms of different neurodegenerative diseases. However, rather than being isolated reports on individual conditions, certain themes have emerged across a number of studies covering diverse diseases and model systems. Accordingly, in the following section, we discuss the use of scRNA-seq to discover new disease-associated cell states, uncover molecular signatures and mechanisms of disease, and to chronicle the emergence of transcriptomic phenotypes across a number of neurodegenerative disorders.

#### 3.3.1 . Disease-associated cell states

An elegant example of where scRNA-seq’s power to discern new cell responses has enhanced disease understanding is in the identification of new disease-associated cell states. The first prominent example of this in the brain was the report of a new microglia state observed in a mouse model of Alzheimer’s disease (AD): the disease-associated microglia (DAM) [103]. This type of microglia was identified by comparing the single-cell profiles of enriched immune cells from the brains of wild-type mice and a mouse model carrying five human familial AD mutations (5xFAD). Using unsupervised clustering analysis to classify the cells, the authors identified a microglia state exclusive to the 5xFAD brains. These cells classified very close to wild-type microglia based on the expression of known markers (e.g. *Cst3, Hexb*), but displayed significant differences in gene expression of typical microglia homoeostatic genes (e.g. *P2ry12*/*P2ry13, Cx3cr1, Tmem1*), known AD risk factors (e.g. *ApoE, Lpl, CD9, Ctsd, Tyrobp, Trem2*), and other genes involved in lipid metabolism and phagocytosis (e.g. *Lpl, Cst7*). Closer analysis revealed two distinct populations, with one representing an intermediary step and the other constituting a more advanced state. Due to their close similarities to homoeostatic microglia, this finding would have been very challenging to make using immunostaining alone. Meanwhile, the gene expression differences that distinguish the closely related populations were masked in bulk RNA-seq due to the low ratio of DAM to homoeostatic microglia (~7%).

Recent GWAS has found numerous risk loci around genes highly, and sometimes uniquely, expressed in microglia. This has therefore triggered much interest in how they are involved in the early stages of AD aetiology and whether they could be viable therapeutic targets [104]. Supporting relevance of DAM to the human condition then, immunostaining with the newly identified DAM marker, *LPL*, revealed that a related human microglia population could be detected in human AD brains but not in control tissue. *LPL*-stained DAM were found to colocalise around a core pathological trait of AD, extracellular amyloid beta (Aß)-plaques, in both the 5xFAD mice and human AD tissue. Further, they appeared around the time of onset of plaque formation and contained Aß particles in the mice [103]. This raises the hypothesis that triggering DAM may be a neuro-protective response that helps clear protease-resistant aggregated proteins. Interestingly then, DAM were also evident in the SOD1 (G93A) mouse model of another neurodegenerative disease associated with perturbed protein clearance, ALS. Urging further investigation though are human-centred snRNA-seq studies that have characterized the transcriptomes of human AD-enriched microglia populations [105,106]. Whilst these confirmed overlap between the mouse and human populations, such as an upregulation of the *APOE* gene [105], they also revealed changes to other AD-linked genes not seen in the animal models (e.g. the complement component gene, *C1QB*, and the pattern recognition receptor, *CD14*), and no change to those tied to DAM progression in mice (e.g. *TREM2*). Meanwhile, AD-associated human profiles partially overlap with the transcriptome-profiles of aged microglia seen in non-AD cases [107]. Thus, whilst human and mice AD-associated microglia states clearly share overlapping features, they still appear distinct from one another and may also be present in aged individuals.

In addition to DAM, disease-associated astrocytes (DAA) have recently been identified in the same 5xFAD mouse model of AD [108]. These DAA express a unique set of genes compared to five other detected astrocyte subclusters. This includes genes involved in endocytosis, complement cascade and ageing (e.g. *Serpina3n, Ctsb*). The new cell type was confirmed exclusively in the AD mice astrocytes by IHC with DAA markers (Gfap, Serpina3n, Vim), were localized adjacent to the Aß-plaques, and expressed higher levels of a serine protease inhibitor linked to compromised amyloid degradation, *Serpina3n*. By profiling the 5xFAD mice in a time-resolved manner, it was revealed that DAA could be detected by four months of age. This is a time-point pre-symptomatic for cognitive decline and suggests they could be an early attempt by the brain to contain the accumulation of misfolded proteins. DAA-like cells were again also evident in aged human brains, and these were found in higher frequencies in individuals with AD. It therefore remains unclear whether their emergence is a universal phenomenon or whether it is amyloid associated. However, as with DAM, there is now much interest in both clarifying and controlling the neuroprotective or neuro-deleterious properties of these DAA at pre-symptomatic time-points to determine whether they could be manipulated for therapeutic benefit in the future.

Beyond AD, disease-exclusive oligodendrocyte lineage cells have been reported in the spinal cord of mice induced with experimental autoimmune encephalomyelitis (EAE), a mouse model of multiple sclerosis (MS) [109]. Specifically, three additional oligodendrocyte precursor cells clusters were identified in the EAE mice compared to their healthy littermates. One of these contained a mix of oligodendrocyte precursor cell state transitions that overlapped both healthy and EAE-exclusive cells, whilst the other two were disease exclusive and characterized by expression of specific genes (e.g. transcription factors *Myrf, Hes1* and *Hes5*). The gene expression profiles confirm that oligodendrocyte precursor cells undergo proliferation and differentiation in EAE, but also suggest a transition to previously unidentified transcriptional states. Five EAE exclusive oligodendrocyte lineage states were also observed: a cluster of differentiation-committed oligodendrocyte lineage precursors, newly formed oligodendrocyte lineage cells, and three mature oligodendrocyte lineage populations. Notably, these had interesting gene expression signatures suggestive of unique roles in the disease that are discussed in the following section. Complementing this murine study, oligodendrocyte heterogeneity has recently been dissected in the adult human brain with snRNA-seq [110]. At least six mature oligodendrocyte populations were characterized based on their unique gene expression profiles that correlated well with the adult mouse counterparts. Similar to the EAE mouse model, specific human oligodendrocyte subtypes showed either increased or decreased representation in the diseased white matter of MS patients. Notably, one specific oligodendrocyte subtype that is lost in MS is predicted to be the normal, fully mature and stable oligodendrocyte population in healthy individuals, whilst the transcriptional signatures of the populations overrepresented in MS appear to have compromised ability to provide metabolic support to neurons. This opens up the intriguing possibility that restoring oligodendrocyte heterogeneity in MS could have therapeutic potential.

#### 3.3.2. Molecular signatures and mechanisms

Further to new cell-state identifications, the gene expression profiles of specific cell populations have been used to interrogate molecular signatures and mechanisms of disease at cell-type resolution, and to help identify putative therapeutic targets. For example, follow-up genetic analysis revealed that the aforementioned AD-associated DAM in mice are generated through a two-step mechanism of activation of homoeostatic microglia, and that the transition to each step is regulated by different genes [103]. Specifically, by searching for potential key regulators from the list of DEGs, the *Trem2* gene, a major risk factor for AD, was found to be increased in the DAM. By using scRNA-seq to subsequently compare DAM abundance in the brains of 5xFAD mice bred on both *Trem2*+/+ and *Trem2*-/- backgrounds, it was found that the *Trem2*-/- mice were completely depleted of DAM and instead accumulated a large number of cells in the intermediate state. This elegantly demonstrated that the homoeostatic microglia progressed through an initial activation via an unknown, *Trem2*-independent mechanism to an intermediate state (stage 1). This intermediate stage can then be further activated to DAM by a secondary activation signal that is *Trem2* dependent (stage 2) and leads to a potentially neuroprotective programme of gene expression that activates phagocytosis and lysosomal degradation pathways. Whilst the molecular trigger of the stage 1 transition remains unknown, the understanding ascertained could imply that appearance of DAM occurs too late or in insufficient numbers to combat AD when particular genetic backgrounds are present (e.g. rare variants of *TREM2*) or during normal ageing. However, given that excessive phagocytosis by microglia can be deleterious to stressed-yet-viable neurons [111], it is also plausible that DAM may alternatively contribute to the disease process. Further experiments to deplete key DAM genes will thus be necessary to confirm whether the DAM are in fact good or bad players in this AD model.

In contrast to the DAM, the molecular triggers of DAA have not yet been determined. However, the use of both nearest-neighbour graphs and transition markers to interrogate origins of different astrocyte sub-populations in the 5xFAD mice has revealed that homoeostatic *Gfap*-low astrocytes similarly progress through an identifiable transitionary step before becoming DAA in what is a dynamic activation process [108]. The progression is also evident in aged mouse brains (>13–14 months), indicating that such changes also occur in normal ageing brains. These observations suggest their activation could in fact be a normal and early gliosis attempt by the brain to contain the accumulation of misfolded proteins. However, their eventual progression to fully established DAA may be associated with a new destructive phase that in fact progresses the disease. Indeed, the DAA express an inflammatory signature alongside genes that inhibit amyloid degradation (i.e. *Serpina3n*). Characterizing the molecular triggers of each stage of activation will thus be important if we are to harness their neuroprotective properties whilst restricting their neurodeleterious properties in the future.

Specifically, in the human condition, two studies have established a cell atlas of differential gene expression changes that occur in AD in both the prefrontal cortex [105] and entorhinal cortex [112]. At a broad level, this revealed all major cell types are affected at the transcriptional level in AD, and that neurons tended to display downregulation of genes whilst neuroglia transitioned to activated states with many genes upregulated [105]. Most changes were then cell-type specific. Among others it was found that endothelial cells upregulate genes involved in cytokine secretion and immune responses (e.g. *HLA-E, MEF2C, NFKBIA*), microglia downregulate homoeostatic genes (e.g. *CX3CR1, P2RY12, P2RY13, DEG4*), genes implicated in cell–cell adhesion (e.g. *CD86, CD83*), lipid responses (e.g. *LPAR6*), and G-protein-coupled receptor pathways (e.g. *GPR183, LPAR6*) [112], whilst *LINGO1*, a negative regulator of neuronal survival, axonal integrity, and oligodendrocyte differentiation, is upregulated in excitatory neurons and oligodendrocytes [105,112]. Meanwhile, snRNA-seq unsurprisingly revealed cell-type-specific DEG signatures with disease relevance which would have been masked in bulk RNA-seq studies. For example, it revealed that different cell types of the same brain region respond differently to the disease. Specifically, the aforementioned *APOE* gene was found upregulated in AD-microglia and downregulated in AD-astrocytes [105]. Together the gene expression changes clarified that almost every cell type had penetrant disease-associated transcriptional changes that could segregate AD cells from control counterparts [112]. Notably, comparing diverse AD cases that were either absent of pathology or had established pathology revealed that most major transcriptional changes across cell-types occurred early in disease progression [105]. However, a more granular look at specific subtypes of various cell populations revealed state changes associated with pathological status. For example, excitatory neuron subpopulation 4, inhibitory neuron subpopulation 0, astrocyte subpopulation 1, and oligodendrocyte subpopulation 0 were isolated from individuals with a high amyloid burden and advanced stage of the disease, whilst excitatory neuron subpopulation 6, inhibitory neuron subpopulation 2, astrocyte subpopulation 0, and oligodendrocyte subpopulation 1 were found in AD cases currently absent of pathology. These subpopulation-specific states appear to underlie the AD-specific transcriptional differences observed at the broad cell-type level. For example, excitatory neuron subpopulation 4 alone could explain the increased *LINGO1, RASGEF1B*, and *SLC26A3* expression associated with the broader excitatory neuron classification.

Whilst cell-type sub-clustering consistently showed that neuroglia separate into healthy and disease clusters more clearly than neurons, thus implying that disease-associated transcriptional changes are perhaps more robust in these cell types, snRNA-seq also revealed more generalized and coordinated differential gene expression responses across multiple cell types in AD in both studies. One such general coordinated differential gene expression cluster changing in all cell types involves the response to topologically incorrect proteins and cell stress. Specifically, there is an increase in the expression of genes encoding molecular chaperones (e.g. *HSP90AA1, BIN1, DNAJA1*) that might be a reaction to protein misfolding [105,112]. It is likely this might be a consequence of the extracellular accumulation of Aß deposition that is both initiated and found in the vicinity of multiple cell types. Further, support cells such as oligodendrocytes, astrocytes, oligodendrocyte precursor cells, and endothelial cells upregulate genes that counteract cell death and which may be part of a neuroprotective drive. In contrast, there is a downregulation of genes involved in behaviour, cognition and synapse organization across neurons, astrocytes, oligodendrocytes and oligodendrocyte precursor cells that is consistent with numerous other studies.

Applying the similar methodology to that used in aforementioned ASD studies [55,82,84,86] these snRNA-seq datasets of human AD tissue have also facilitated important interrogation of cell-subtype specific expression patterns of ~1000 AD-associated GWAS genes [112]. This unexpectedly revealed that many AD-associated genes display specific expression in microglia to further emphasize the central role of these cells in AD (e.g. *NPP5D, HLA−DRB5, PLCG2, RIN3, TBXAS1*). Others revealed previously unknown expression patterns across different cell types (e.g. *KCNN3* in astrocytes, *MYT1* in oligodendrocytes). However, the granular assignment of individual transcriptomes to different cell sub-classes also allowed more complex expression specificity patterns to be uncovered. Among others, the previously considered oligodendrocyte-specific gene, *ADAMTS18*, was found upregulated in AD-associated astrocytes, oligodendrocytes, and oligodendrocyte precursor cells, yet downregulated in neurons. Meanwhile, *APOE* was found to be downregulated in specific oligodendrocyte [112] and astrocyte [105,112] subclusters, yet upregulated in a single microglia subcluster. Whilst the link between *APOE* alleles and AD microglia is well established, its association with oligodendrocytes is novel. It will now be important to determine whether the loss of endogenous *APOE* expression in oligodendrocytes plays a role in the poorly understood myelination changes observed in AD [113].

Like AD, scRNA-seq has already been useful to ascribe previously unrecognized functions for oligodendrocytes in MS. Studies using bulk RNA-seq pointed at peripheral immune system cells and brain microglia to be the most susceptible cell types in MS, but the MS susceptibility genes are also found in EAE exclusive oligodendrocyte precursor cells, oligodendrocyte lineage cells and mature oligodendrocytes. Accordingly, scRNA-seq revealed that DEGs in the oligodendrocyte precursor cells and oligodendrocyte lineage cells of EAE mouse models were related to immunoprotection, interferon response pathway, antigen processing and presentation via major histocompatibility complex class I (MHC-I) and II (MHC-II). These were all previously thought restricted to microglia and macrophages in MS, and instead suggested that those cells might in fact be targeted during the disease despite not expressing myelin proteins [109]. Human MS oligodendrocytes were similarly confirmed to express adaptive immunity proteins. Thus, the validated changes implied certain populations of oligodendrocyte precursor cells and oligodendrocyte lineage cells have the capacity to mount an immune response. Co-culture experiments subsequently demonstrated the increased expression of MHC-II genes can be triggered in these cells by EAE-specific T-lymphocytes, whilst exogenous activation of the response confirmed that oligodendrocyte lineage cells acquire the ability to both phagocytose myelin and trigger naive T lymphocytes (both memory and effector). The findings have important ramifications for disease understanding, as oligodendrocytes were previously considered a passive victim of an autoimmune dysfunction initiated in the peripheral immune system. Instead, the data suggest that oligodendrocyte lineage cells could play an active role as immunomodulators of the disease, and raises hope that influencing this property may have therapeutic potential in the future.

#### 3.3.3. Disease progression

A caveat of comparing cell transcriptomes from samples at single disease/developmental stages is that it implies a previous knowledge of the disease progression. Yet cells in different disease states express a unique transcriptome and undergo transcriptional reprogramming as they move from one stage to another. One logical option to study disease progression is therefore to directly compare scRNA-sequencing datasets from samples at different time points of the disease in order to chronicle such changes. For example, interrogation has been carried out of both hippocampal and cortical microglia at multiple time points of the inducible CK-p25 mouse model that phenocopies neurodegeneration. This revealed different stage-specific states of neurodegeneration-associated microglia, rather than a completely new microglia type [77]. Indeed, healthy microglia transitioned to late-response microglia through two transient states, each of them containing cells in different phases of the cell cycle (G1/S and G2/M). Together these implied that microglia show an early increase in proliferation in response to the induced neurodegeneration. Meanwhile, there was a heterogenous overexpression of immune-related genes and enrichment of binding motifs of the interferon-regulatory factor family in the sequence of the DEGs of the late-response. This demonstrated that these microglia were mounting a pronounced immune response during the later stages of neurodegeneration, and also that two molecularly distinct reactive microglia phenotypes could be distinguished. One of these had high levels of antiviral and interferon genes and the other with high levels of MHC class II genes. Further analysis of the transcriptional dynamics revealed the emergence of temporally distinct subsets of these immune response-related genes as the microglia progressed; certain chemokines (e.g. *Ccl3, Ccl4* and *Cxcl16*) and inflammatory cytokines (e.g. *Mif*) were upregulated at early stages following neurodegeneration whilst other immune-related genes (e.g. *H2-D1, Axl, Apoe*) were upregulated late.

This study demonstrates the benefits of a time-resolved study when feasible. However, this is both challenging in human cases where time-resolved samples from the same individuals are non-trivial to obtain, whilst it can also increase experimental costs due to the need for increased sample profiling. It is therefore important to appreciate that neurodegeneration is an asynchronous process within a tissue: at a given time, each cell captured in a scRNA-seq dataset can provide a snapshot into the transcriptional reprogramming process along its intended trajectory. Accordingly, a so-called ‘pseudotime’ analysis of scRNA-seq datasets can allow an alternative type of study of disease progression within a single sample in an unbiased manner. To achieve this, cells are generally represented as points in a diffusion map and trajectories are built between individual cells or cell clusters in a way that minimizes gene expression changes between neighbouring cells along the reprogramming route. Using all points, the most probable path between two points is then calculated to define the trajectory or pseudotime. Several trajectory inference analysis or pseudotime ordering algorithms have subsequently been developed to define gene expression changes in disease progression from scRNA-seq datasets, each with their own advantages and disadvantages [114]. These typically use gene abundance to identify the nearest neighbours, although the ratio of unspliced and spliced mRNAs can even be used to estimate the time derivative of gene expression and predict future states of individual cells [115].

Using this pseudotime concept, a modified version of the Wanderlust algorithm [71] was used to make the aforementioned discovery that AD-associated DAM not only constitute a single new cell type but actually two subtypes of microglia; an intermediary population and a more advanced *Trem2*-dependent form [103]. Each brain then contains microglia at different progression stages of the two-step progression. However, Wanderlust is only able to identify linear trajectories and requires a manual definition of the starting (homoeostatic microglia) and the end point (DAM) of the pseudotime to order the other cells in the points between them. Accordingly, the use of this particular algorithm required *a prior* knowledge of the disease progression and relied on previously identified marker genes for a guided analysis. Other algorithms such as Monocle [70] organize single cells in a time-line corresponding to a biological process. Monocle achieves this by first classifying cells into clusters corresponding to cell types or cell state by either using known marker genes or with unsupervised clustering. Using this semi-supervised pseudotime analysis, it has instead been reported that microglia from the prefrontal cortex of a related *App* knock-in mouse model of AD in fact differentiate into multiple mutually exclusive states as pathology emerges instead of following the linear trajectory implied by Wanderlust (i.e. activated response microglia, interferon response microglia and transiting response microglia) [116]. These microglia states can all be found in the same brain, and the activated response microglia importantly show overlapping gene expression signatures to the DAM previously identified by Keren-Shaul and colleagues [103]. Based on these findings, the study posits that there are no ‘new’ microglia states in sick brains, but rather a quantitative redistribution of the ratio of each microglial type in disease. This is thus akin to the quantitative redistribution of oligodendrocytes observed in MS [110]. Notably, the most enriched AD microglia state from this *App* knock-in mouse model was also enriched in healthy aged brains [116]. This therefore suggests that the term, DAM, may be an overstatement. It will be important to see if this is also the case for DAA in future studies.

In addition to complex multicellular tissues, pseudotime analysis can also be applied to cultured cells to chronicle the emergence of molecular disturbances. This is particularly useful for patient-derived iPSCs that have emerged as a viable option to study neurodegenerative disease progression. Similar to a tissue, even within an enriched monoculture, pseudotime analysis of individual cells can reveal differences relevant to disease progression that would remain undetected in studies of whole cell populations [117]. As an example of this, scRNA-seq data from hiPSC-derived dopamine neurons from healthy and Parkinson’s disease (PD) patients with mutations to the *GBA* gene have been used to study the progression of molecular phenotypes using the Ouija algorithm, a semi-supervised method that uses expression switches of a small set of predefined marker genes to infer the pseudotime trajectory [118,119]. Specifically, the assessed trajectory was defined using 60 genes that had previously been found to be differentially expressed in both bulk RNA-seq and scRNA-seq datasets comparing control lines to lines of PD GBA-N370S cases. Pseudotime analysis classified these genes as either early-disease genes (e.g. *TSPAN7, ATP1A3, RTN1* and *PRKCB*) or late-disease genes (e.g. *ERO1A, FKBP9* and *PDIA6*) for switching their expression in either early or late stages of the disease pseudotime, respectively. Moreover, the analysis identified the epigenetic modifier, HDAC4, as a potential target for Parkinson’s disease (PD) treatment. Indeed, the aforementioned early-disease genes are targets of HDAC4 and are downregulated in the early stages of the pseudotime trajectory, although the expression of HDAC4 itself was not changed. It was subsequently confirmed that HDAC4 showed increased localization in the nucleus that was consistent with the downregulation of its targets. Notably, pharmacological treatment of the PD hiPSC lines with three different FDA-approved compounds that inhibit HDAC4 localization showed correction of all four HDAC4-controlled genes, and ameliorated increases in other genes associated with late-stage ER stress of GBA-mutated lines. Moreover, two treatments also improved previously observed perturbations to autophagic and lysosomal pathways in the PD iPSCs, and reduced associated increases in an extracellular alpha-synuclein release. Thus, scRNA-seq data in combination with in vitro experiments has excitingly raised the possibility of FDA-approved HDAC4 inhibitors being therapeutic for PD treatment.

As a final pseudotime development, the SCN3E [120] and Monocle 2 [70,121] algorithms, unlike Wanderlust or Ouija, allow prediction of more complex ‘branched’ trajectories, rather than just linear progressions. Their subsequent application with data from MS post-mortem tissue has allowed oligodendrocytes to be subdivided into different cell states as they progress from oligodendrocyte precursor cells to two mutually exclusive final states via a tree-like trajectory. Contrary to mice models then, this implies that there is indeed no new disease-associated cell population in human MS, rather an imbalance between cell states. Specifically, there are fewer nuclei in the oligodendrocyte precursor cell state, reductions of one of the end point subclusters and an intermediate cell state on its pathway, and an enrichment of the second end point subcluster [110]. Importantly, identified cell-state markers were used for immunohistochemistry on post-mortem MS tissue to confirm the scRNA-seq results and map the spatial characteristics of these cells in the brain. The genes associated in the pseudotime trajectory suggest that the intermediate subclusters in both pathways represent actively myelinating oligodendrocytes. Moreover, transcriptome analysis for each end point subcluster revealed that several myelin protein genes were upregulated in mature oligodendrocytes in MS. These findings suggest a re-evaluation of current strategies to treat MS is required. Currently, efforts are made to increase differentiation from oligodendrocyte precursor cells to mature oligodendrocytes. However, this likely isn’t enough as the right proportion of both mature oligodendrocyte populations may also need to be reached.

## 4. Future perspectives of scRNA-seq in neuroscience

It is clear that impressive progress has been made over the last decade using scRNA-seq to enhance our understanding of the cellular interplay of the brain in both health and disease. However, with the scRNA-seq methods entering a seemingly mature phase, there remain certain limitations of the approach and promising applications which remain beneficial to address. Since a core requirement of scRNA-seq is the preparation of single cells in suspension, a major caveat is that scRNA-seq suffers from an immediate loss of spatial resolution upon sample processing. Accordingly, whilst scRNA-seq datasets inform what happens in the cells and the molecular relationship between them, we do not know whether cells are close together or far apart in the original samples, nor whether they are proximal to pathologies or associated with specific tissue architecture. Spatial context has been regained in the past by retrospectively carrying out *in situ* labelling of cell-type markers identified from scRNA-seq datasets. For example, in its simplest and lowest-throughput form, this has confirmed the proximity of DAM around amyloid plaques in AD transgenic models [103]. However, integration of many *in situ* marker probes with machine learning methods has the potential to spatially reconstruct whole scRNA-seq datasets [122–124].

An emerging high-throughput alternative is the integration of the complementary technology to scRNA-seq, spatial transcriptomics [125]. Here, RNA is released from tissues sections and captured by the spatially ordered and barcoded probes that lie beneath. On-bead reverse transcription is then followed by pooling, cDNA library synthesis and sequencing. The subsequent barcode reads accordingly allow individual mRNAs to be matched back to their spatial location in the original section. The method has now been applied to numerous tissues [124,126,127] including the CNS [128,129], whilst reducing probe-to-probe distance has steadily improved spatial resolution from study-to-study [130]. However, cells rarely align perfectly to the ordered probes such that single-cell resolution is incomplete at an individual probe level. At 10 μm probe resolution, ~65% of probes align to a single mouse hippocampal cell [131]. At 2 μm probe resolution, ~50% of probes align single mouse olfactory bulb cells [130]. Leveraging paired haematoxylin and eosin (H&E) stained images of samples to identify nuclei overlapping multiple probes can allow the sharing of information from a probe’s nearest neighbour to improve the single-cell assignment. This leads to ~ 60% of coordinates being assigned to a single-cell type when using a 2 μm probe resolution [130]. Whether granular dissection of closely related cell populations can be achieved like with scRNA-seq remains unclear though, thus making it unlikely to eliminate the need for scRNA-seq in the future. Instead, computational methods have recently emerged to facilitate the integration of scRNA-seq and spatial transcriptomics datasets. Particularly noteworthy here, the Slide-seq variant of spatial transcriptomics assembles cost-effective homemade slides using mRNA-capture beads similar to those used in scRNA-seq [131]. Following the on-slide sequencing of bead indexes to determine barcode coordinates, spatial transcriptomics are next performed like in other workflows. Notably, in an elegant proof-of-principle, the computational integration of Slide-seq and scRNA-seq datasets from sequential sections of mouse brain tissue has impressively allowed an accurate 3D-spatial contextualization of scRNA-seq profiles, and the subsequent assembly of a cell-resolution tissue map. Accordingly, this study, alongside related reports from alternative [124] tissues, make it clear that spatial transcriptomics will become an important complement to the scRNA-seq field in the future as we extend into cell-resolution 3D maps of CNS tissue in both health and disease.

Last, studies of neurological conditions have the ultimate goal of understanding underlying mechanisms such that they can then be leveraged to treat patients via preventing, stalling or even reversing the condition. It is hoped that future scRNA-seq studies will thus uncover both condition- and genotype-specific molecular mechanisms that can be used to develop effective and well-rationalized therapies. In this context, time-resolved studies, pseudotime analysis and the reconstruction of cell-type-specific gene regulatory networks will be extremely important areas of future scRNA-seq research in the neurosciences. Indeed, our current understanding of the pre-symptomatic cellular phases of neurological conditions remains severely limited, whilst the monitoring of potential therapies is often restricted to an analysis of a single-cell type or the heterogeneous tissue as a whole. Chronicling the earliest molecular dysfunctions across cell populations has potential to reveal the most tractable opportunities for developing effective therapies targeting condition initiation, whilst monitoring beneficial and detrimental responses of treatments across all cell populations over time could facilitate refinements to secure the best clinical outcomes. Meanwhile, the reconstruction of cell-type-specific gene regulatory networks has the potential to identify regulatory proteins driving cell state transitions and key differential expression changes in the CNS in both health and disease [26,27,86,112]. Although time-resolved study is challenging with samples from human patients, it is readily applicable in research studies or pharmacological trials which use animal models [103] or patient-derived stem-cell models [118]. The aforementioned time-resolved characterization of DAM [103] and DAA [108] transition states and identification of *TREM2* as a key gene for DAM establishment elegantly evidence this, and have subsequently rationalized on-going development of therapies for manipulation of these cell states for clinical benefit.

## 5. Summary

The maturation of scRNA-seq as technology has clearly been a welcome addition to the toolkit for studying RNA metabolism across the life sciences. Indeed, the technology allows complex cellular systems to be broken down into their individual cellular components for high-resolution study of cell populations. This has been transformative in the immensely complex CNS. Indeed, scRNA-seq has already been leveraged to expand our cellular characterization of the CNS (Table 2, Fig. 2), and to elucidate the cell-type-specific responses and molecular mechanisms that occur in various neurodevelopmental and neurodegenerative conditions, such as ASD, AD and MS. In future it will be important to develop scalable workflows for whole transcriptome sequencing such that all cellular transcripts and isoforms can be assessed at cell resolution, and to continue integration with other single cell ‘omic’ technologies and complementary spatial transcriptomics workflows to provide increasingly holistic understanding. Together this will facilitate evermore ambitious studies aimed at clarifying the underlying mechanisms of neurodevelopment and neurodegeneration, and help develop well rationalized therapeutics for the myriad neurological conditions in the future.
